# Bio-Inspired Autonomous Learning Algorithm With Application to Mobile Robot Obstacle Avoidance

**DOI:** 10.3389/fnins.2022.905596

**Published:** 2022-06-30

**Authors:** Junxiu Liu, Yifan Hua, Rixing Yang, Yuling Luo, Hao Lu, Yanhu Wang, Su Yang, Xuemei Ding

**Affiliations:** ^1^School of Electronic Engineering, Guangxi Normal University, Guilin, China; ^2^College of Innovation and Entrepreneurship, Guangxi Normal University, Guilin, China; ^3^Department of Computer Science, Swansea University, Swansea, United Kingdom; ^4^School of Computing, Engineering and Intelligent Systems, Ulster University, Derry, United Kingdom

**Keywords:** neuroanimats, spiking neural networks, reinforcement learning, spike-timing-dependent plasticity, robot

## Abstract

Spiking Neural Networks (SNNs) are often considered the third generation of Artificial Neural Networks (ANNs), owing to their high information processing capability and the accurate simulation of biological neural network behaviors. Though the research for SNNs has been quite active in recent years, there are still some challenges to applying SNNs to various potential applications, especially for robot control. In this study, a biologically inspired autonomous learning algorithm based on reward modulated spike-timing-dependent plasticity is proposed, where a novel rewarding generation mechanism is used to generate the reward signals for both learning and decision-making processes. The proposed learning algorithm is evaluated by a mobile robot obstacle avoidance task and experimental results show that the mobile robot with the proposed algorithm exhibits a good learning ability. The robot can successfully avoid obstacles in the environment after some learning trials. This provides an alternative method to design and apply the bio-inspired robot with autonomous learning capability in the typical robotic task scenario.

## 1. Introduction

The human brain is a complex system that has billions of neurons and trillions of synapses (Azevedo et al., 2009). The number of neurons is so large that it is a challenge to know what happens in the brain, especially the connection between the microscopic properties of neurons and macroscopic biological behavior. However, based on the studies of the biological nervous system, researchers still get a lot of inspiration from them, i.e., Artificial Neural Networks (ANNs). ANNs are programming paradigms inspired by the biological nervous system, which allows computers to learn from observation data and has been used to solve various problems. Spiking Neural Networks (SNNs) are often called the third generation ANNs and focus on the accurate simulation of biological network behavior, and provide a promising computing paradigm due to their biological basis (Wang et al., 2010). For example, a novel spike-based continual meta-learning called meta-learning with minimum error entropy is proposed by Yang et al. (2022b), where the experimental results show that the scheme has good performance in three experiments, including autonomous navigation, robust working memory in the store-recall task and robust meta-learning capability for the sequential MNIST data set. In Yang et al. (2022a), a novel self-adaptive multicompartment neuron model is proposed, and a recurrent SNN architecture, along with spike-driven learning algorithms in supervised and meta-learning frameworks is constructed based on this neuron model. This scheme also performs well in agent navigation and meta-learning of MNIST classification tasks.

The robot controlled by biological neurons is often called the neuroanimats, where animat represents “animal” and “automat” (Chicca et al., 2014). The target is that a robot learns by using SNNs, and its behavior is the same as an animal in a real-world environment. Since the concept was first proposed by Brand et al. (1995), many successful neuroanimats cases have been reported. A tactile robot called CARL-SJR is constructed in the approach of Chou et al. (2015). CARL_SJR can feel the touch of the real world and change the color of its surface to respond to different types of touch. In Rothman and Silver (2014), a crocodile robot and its control bracelet are reported. The control bracelet can detect hand gestures *via* myographic signals and send corresponding commands to the crocodile robot. Besides, the crocodile robot can learn from the environment and reproduce crocodiles' basic behavior, such as searching and “eating” an object. In Lobov et al. (2020), a LEGO robot controlled by SNN is proposed. The robot can learn to avoid obstacles and relearn when the environment changes. These cases are relatively successful, however, the potential of neuroanimats is still not fully exploited due to two aspects (Bing et al., 2019b): (1). Unlike the second generation, ANNs with backpropagation algorithms and deep learning approaches, SNNs lack effective learning algorithms. The main problem faced by the researchers building neuroanimats is the absence of learning algorithms (Lobov et al., 2020). In a more general context, the learning principles of biological neural networks are not explored up to a sufficient level for designing engineering solutions (Gorban et al., 2019). (2). Software simulation is a common way to implement SNNs, but for complex SNNs, computational cost greatly increases the processing time and affects the real-time performance of the networks (Brette et al., 2007; Shayani et al., 2008; Kulkarni et al., 2017). Besides, neuroanimats need to explore and learn in the real world, so their power consumption is also needing to be considered. The demand for high performance and low power consumption makes the general processor unable to meet the needs.

To solve these problems, the following two works are carried out in this article. (1) The existing algorithm reward modulated Spike-Timing-dependent Plasticity (STDP) is improved to match the macroscopic biological behavior observed by existing studies. Subsequently, an SNN with psychologically plausible at the macroscopic level and biologically plausible at the microscopic level is designed for mobile robot obstacle avoidance based on the improved algorithm. Finally, the effectiveness of the designed SNN was deployed on a mobile robot for verification. Besides, a biologically plausible rewarding generation mechanism is used to generate the reward signals. With the help of this mechanism, the release of reward signals is controlled by special dopamine neurons rather than simple function mapping. (2) For the SNN with a high energy consumption ratio and simple implementation on hardware characteristics (Liu et al., 2018; Yang et al., 2020, 2021), combined with the application scenario of mobile robot, the proposed SNN is implemented by using Field Programmable Gate Array (FPGA). It improves the ability of data processing of the mobile robot and provides a hardware system for future expansion.

The rest of this article is organized as follows. Neuron models, synapse models, learning methods, SNN's architecture, and hardware platform of the mobile robot are presented in Section 2. Experimental results are given in Section 3. Section 4 is the discussion, and Section 5 is the conclusion.

## 2. Materials and Methods

In this section, the neuron models, synapse models, and learning methods are presented. It will start by introducing Integrate-and-Fire (IF) neuron model, Leaky Integrate-and-Fire (LIF) neuron model, and synapse model. Then, the STDP and reward modulated STDP will be introduced. From this, the autonomous learning algorithm based on reward modulated STDP and biological plausible rewarding generation mechanism are proposed. All the parameters used in the above sections can be found in Table 1. Finally, the SNN's architecture and hardware platform of the mobile robot are presented.

**Table 1:** The parameters used in Spiking Neural Network (SNN) models and learning algorithm.

**Parameter**	**Parameter description**	**Value**
*R*_*IF*_	Membrane resistance of IF neuron	1 MΩ
τ	Membrane time constant of IF neuron	1 ms
*R*_*LIF*_	Membrane resistance of LIF neuron	1 MΩ
τ_*m*_	Membrane time constant	5 ms
*v*_*rest*_	Resting potential	0 mV
τ_*s*_	Synapse current time constant	10 ms
*A*_+_	Positive amplitude of STDP	1
*A*_−_	Negative amplitude of STDP	–1
τ_+_	Pre-synaptic spike trace time constant	10 ms
τ_−_	Post-synaptic spike trace time constant	10 ms
τ_*c*_	Eligibility trace time constant	10 ms
*l*	Constant of learning rate	0.00003
*w*_0_	Initial weight	1
τ_*r*_	Reward signal time constant	2 ms
*C*_*r*_	Constant of reward signal	0.07

### 2.1. Neurons and Synapse Models

Neurons are the elementary processing unit in the nervous system of animals and play an important role in SNNs. According to Izhikevich (2004), there are more than ten kinds of commonly used neuron models. The difference between these neuron models is the level of detail in describing the behavior of biological neurons (Liu et al., 2019a,b). Since the purpose of using neurons in this article is to verify the effectiveness of the proposed algorithm, two types of simple neuron models are chosen (Lu et al., 2021). The first type is the IF neuron model, which is used to encode external environment information of mobile robots into spike trains. The dynamics of a single IF neuron can be described as


(1) $$\begin{array}{l} \tau \frac{dv_{IF}}{dt}=v_{rest}+R_{IF}I_{IF}\left(t\right), \end{array}$$

where τ is the time constant, *v*_*IF*_ is the membrane potential, *v*_*rest*_ is the resting potential, *R*_*IF*_ is the membrane resistance and *I*_*IF*_(*t*) is the injected current. The second type is the LIF model which is used for decision making and generating reward signals. It can be considered as an IF model with a leaky term and can be expressed as


(2) $$\begin{array}{l} \tau_{m}\frac{dv_{LIF}}{dt}=-\left(v_{LIF}-v_{rest}\right)+R_{LIF}I_{LIF}\left(t\right), \end{array}$$

where τ_*m*_ is the membrane potential time constant, *v*_*LIF*_ is the membrane potential, *v*_*rest*_ is the resting potential, *R*_*LIF*_ is the membrane resistance and *I*_*LIF*_(*t*) is the injected current. Neuron models used in this paper have the same threshold voltage of 30 mV and resting potential *v*_*rest*_. When the membrane potential of a neuron reaches the threshold voltage, a spike is fired immediately and the membrane potential is set to *v*_*rest*_. Then the LIF neuron enters a refractory period lasting 2 ms. IF model used in this article has no refractory period.

In SNNs, the transmission of information between neurons is carried out through synapses. The synapse model used in this article has the shape of an alpha function and is often called the alpha synapse, which is used in Destexhe et al. (1994) and Gabbiani et al. (1994). The dynamics of a synapse can be described as


(3) $$\begin{array}{l} \tau_{s}\frac{dI_{syn}}{dt}=-I_{syn}+\overset{n}{\underset{i=1}{\sum }}w_{ij}\delta \left(t-t_{i}\right), \end{array}$$

where τ_*s*_ is the time constant of synapse current, *I*_*syn*_ is the synapse current, *n* is the number of spikes fired by pre-synaptic neuron *i*, δ(*t*) is the Dirac delta function, *w*_*ij*_ is the synaptic weight between pre-synaptic neuron *i* and post-synaptic *j*, and *t*_*i*_ is the fire time of pre-synaptic neuron.

### 2.2. Spike-Timing-Dependent Plasticity

**Figure 1:**
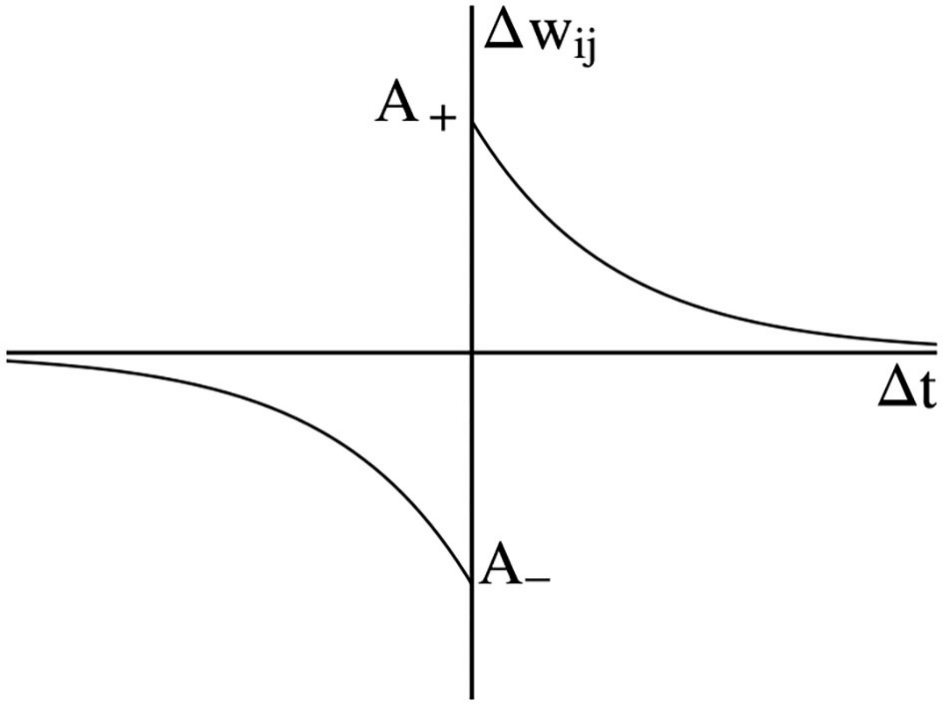
Standard Spike-Timing-dependent Plasticity (STDP) curve.

Spike-Timing-dependent Plasticity is often considered a variant of the Hebbian learning rule and has been proven to have a biological basis. The Hebbian learning rule is that if neurons are activated at the same time, the connection between them will increase. Conversely, if neurons always cannot be activated synchronously, the connection between them will be weakened (Markram, 2011). Consider that two neurons are connected by one synapse, the pre-synaptic neuron *i* fires spike at time $t_{i}^{f}$ and post-synaptic neuron *j* fires spike at time $t_{j}^{f}$, then the change of weight Δ*w*_*ij*_ is a function of time interval $\Delta t=\;t_{j}^{f}-t_{i}^{f}$. The standard form of STDP is shown in Figure 1, where *A*_+_ and *A*_−_ are the amplitude. The weight change when the pre-synaptic spike or post-synaptic spike is fired. If Δ*t*>0, the weight will be increased. If Δ*t* < 0, the weight will be decreased.

**Figure 2:**
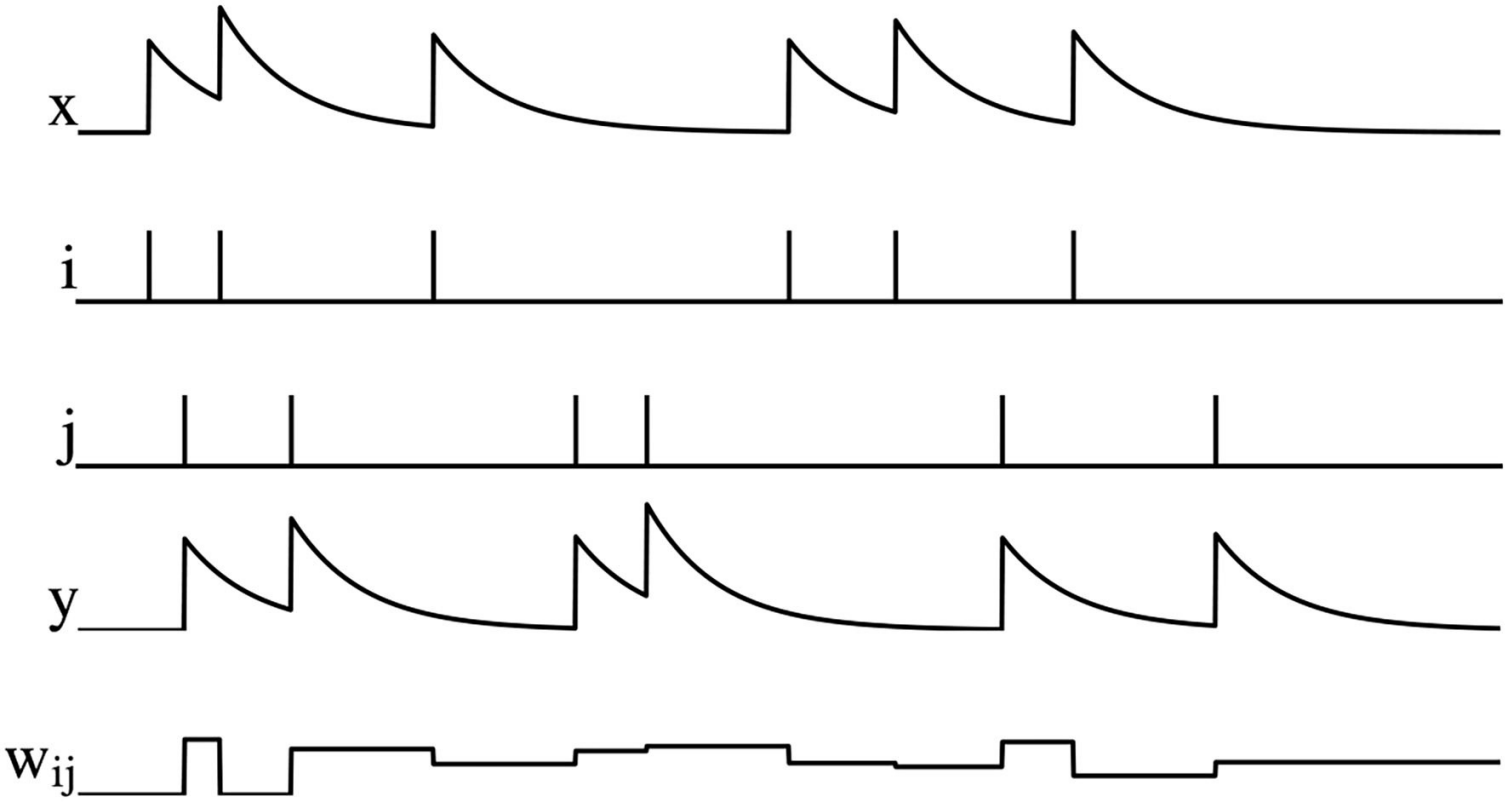
Illustration of PSTDP, where *x* is the pre-synaptic spike trace, *i* is the spikes of pre-synaptic neuron, *j* is the spikes of post-synaptic neuron, *y* is the post-synaptic pike trace, and *w*_*ij*_ is the synaptic weight.

However, all possible pairs of pre-synaptic and post-synaptic spike times need to be considered if this process is implemented plainly, this will consume lots of computing resources, especially in hardware implementation (Mahadevuni and Li, 2017). To solve this problem, pair-based STDP (PSTDP) which proposed by Morrison et al. (2008) is used in this article, which can be described as


(4) $$\begin{array}{l} \{\begin{array}{c} \frac{dx}{dt}=-\frac{x}{\tau_{+}}+\sum_{f}\delta \left(t-t_{i}^{f}\right)\\ \frac{dy}{dt}=-\frac{y}{\tau_{-}}+\sum_{f}\delta \left(t-t_{j}^{f}\right) \end{array}, \end{array}$$

where τ_+_ and τ_−_ are the time constant, δ(*t*) is the Dirac delta function, $t_{i}^{f}$ and $t_{j}^{f}$ are the firing time of pre-synaptic and post-synaptic neurons, respectively. Then the weight change of the synapse is calculated by


(5) $$\begin{array}{l} \frac{dw_{ij}}{dt}=A_{-}y\left(t\right)\underset{f}{\sum }\delta \left(t-t_{i}^{f}\right)+A_{+}x\left(t\right)\underset{f}{\sum }\delta \left(t-t_{j}^{f}\right), \end{array}$$

where *A*_−_ and *A*_+_ are the amplitude. The graphical representation of PSTDP is shown in Figure 2, *w*_*ij*_ decreases when the pre-synaptic spike arrives, otherwise *w*_*ij*_ increases.

### 2.3. Reward Modulated STDP

**Figure 3:**
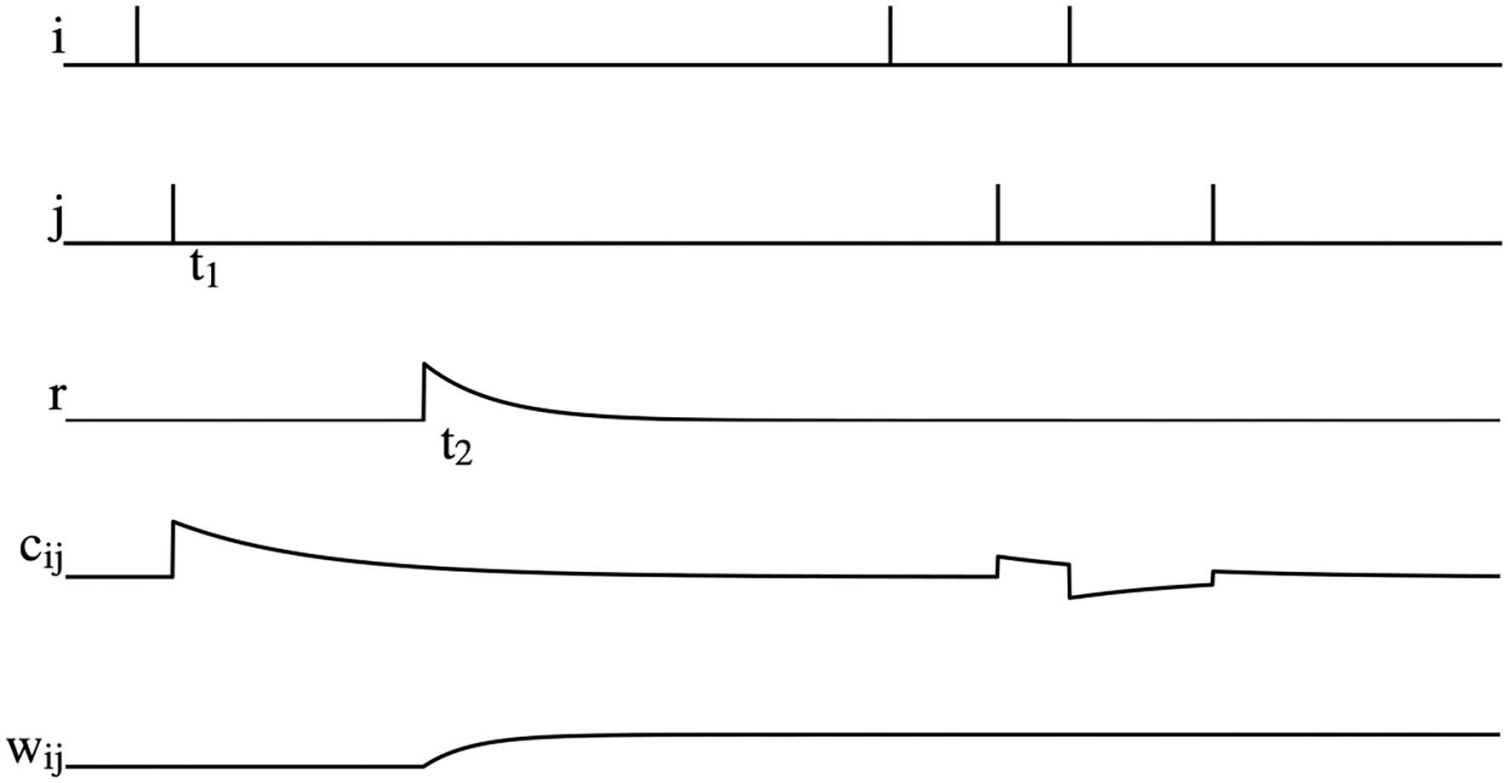
Illustration of reward modulated STDP, where *i* is the spike fired by the pre-synaptic neuron, *j* is the spike fired by post-synaptic neuron, *r* is the reward signals, *c*_*ij*_ is the eligibility trace, and *w*_*ij*_ is the synaptic weight.

Reward modulated STDP, also called dopamine modulated STDP, is a learning algorithm that combines reinforcement learning and STDP. In reward modulated STDP, the influence of pre-and post-synaptic spikes is not immediately reflected in weight but collected by eligibility trace (Florian, 2007; Izhikevich, 2007). Eligibility trace can be seen as a memory of the pre-and post-synaptic spike. Besides, weight change also is affected by reward signals. The weight can only be changed when there are reward signals. The weight dynamics of reward modulated STDP can be described as


(6) $$\begin{array}{l} \frac{dw_{ij}}{dt}=\gamma r\left(t\right)c_{ij}\left(t\right), \end{array}$$

where *r*(*t*) is the reward signals, γ is the learning rate and *c*_*ij*_ is the eligibility trace. The dynamics of eligibility trace can be expressed as


(7) $$\begin{array}{l} \frac{dc_{ij}}{dt}=-\frac{c_{ij}}{\tau_{c}}+STDP\left(\Delta t\right)\delta \left(t-t_{ij\;}\right), \end{array}$$

where τ_*c*_ is the time constant of eligibility trace, *STDP*(Δ*t*) is the STDP term mentioned in Equation 5 and *t*_*ij*_ is the fire time of the pre- or post-synaptic spike. The graphical representation of reward modulated STDP is shown in Figure 3. As can be seen, postsynaptic neuron *j* fires a spike at time *t*_1_, which causes an increase of the eligibility trace *c*_*ij*_ immediately. However, weight *w*_*ij*_ does not change at this time due to the absence of reward signals. At the time *t*_2_, a positive reward signal is released and the weight *w*_*ij*_ begins to increase. After *t*_2_, even if there are spikes fired by the pre- and post-synaptic neurons, *w*_*ij*_ is not changed.

### 2.4. The Improved Algorithm

Reward modulated STDP can explain how behavioral relevant adaptive changes in complex neural networks could be achieved in a self-organizing manner through local synaptic plasticity (Legenstein et al., 2008). At present, the reward modulated STDP algorithm updates the weights by providing the reward signal on demand during the training process and once the training is completed, the reward signals are no longer provided. This is similar to the approaches of Shim and Li (2017) and Chou et al. (2015). However, in biological neural networks, the generation of reward signals is determined by the stimulation of the external environment and the internal network activities (Schultz et al., 1997), so the above process may not be consistent with the biological basis. In addition, existing studies show that reward signals may play an important role in the decision-making process, e.g., the main cause of Parkinson's disease is the decrease of dopamine due to the death of dopaminergic neurons (Freed et al., 2001). Dopamine activity is also an important factor in schizophrenia (Davis et al., 1991). Therefore, this article makes the following two changes to the algorithm based on the assumption that the reward signals are involved in the decision making, i.e., the weights that decay over time and the learning rate affected by the time. The former is derived from Kohonen (1988), often referred to as the forgetting process. The latter is still consistent with the standard reward modulated STDP (in its original form γ can be a function related to time). Then the new algorithm can be described as


(8) $$\begin{array}{l} \frac{dw_{ij}}{dt}=\gamma \left(t\right)r\left(t\right)c_{ij}\left(t\right)-\frac{w_{ij}-w_{0}}{\tau_{w}}, \end{array}$$

where γ(*t*) is the learning rate, *r*(*t*) is the reward signals, *c*_*ij*_(*t*) is the eligibility trace, τ_*w*_ is the time constant of weight and *w*_0_ is the initial weight. The dynamic characteristics of γ(*t*) can be described as


(9) $$\begin{array}{l} \frac{d\gamma }{dt}=l\cdot r\left(t\right)c_{ij}\left(t\right) \end{array}$$

where *l* is the constant. Since the weight decay over time in the absence of reward signals, long-term memory of learning is no longer retained in the weights but is preserved in the learning rate. The learning rate can memorize the process of weight change, if weight is increased, then the learning rate will also be increased. This will lead to more increases in the weights with the same reward stimulus. This design is also consistent with the Hebbian learning rule, i.e., if neurons are activated at the same time, the connection between them will increase.

### 2.5. The Release of Reward Signals

The release of reward signals is important for SNNs and the generation of reward signals is usually a function that directly converts environmental information into reward values. For example, in Bing et al. (2019a), to perform the target tracking task, the reward value is set as a function of the difference between the target and the center position in the image sensor. In Ozturk and Halliday (2016), to map spatial-temporally encoded patterns in spiking neurons, the reward value is set as a function of normalized measure between the actual and desired spike trains for the output neuron. However, in the brain the reward signal is generated within the biological neural network, i.e., it is released by dopaminergic neurons. Therefore, in this section, a biologically plausible reward generation mechanism is designed. Dopaminergic neurons are the neuron that controls the release of dopamine and exist in the mammalian brain (Tobler, 2005). According to Schultz et al. (1997), dopaminergic neurons will maintain a baseline level of activity in the absence of external stimuli. If dopaminergic neurons are stimulated, e.g., the change in environment, then the activity of them will increase, i.e., the average number of action potentials they fired per unit of time will increase. Finally, when the stimuli wear off, the activity of dopaminergic neurons will quickly return to the baseline level. To simulate this process, the LIF neuron model is used. The only change is when the membrane potential of a dopaminergic neuron reaches the threshold voltage, a reward signal is released rather than a spike. Then the reward signal decay exponentially. From this, all reward signals existing in the proposed SNN can be expressed as


(10) $$\begin{array}{l} \frac{dr}{dt}=-\frac{r}{\tau_{r}}+C_{r}\overset{n}{\underset{i=0}{\sum }}\delta \left(t-t_{i}\right), \end{array}$$

where *r* is the reward signals that exist in the proposed SNN, τ_*r*_ is the time constant of reward signals, *C*_*r*_ is the constant, *n* is the total number of reward signals released, δ(*t*) is the Dirac delta function, and *t*_*i*_ is the release time of *i*^*th*^ reward signal.

### 2.6. SNN Architecture

To verify the effectiveness of the algorithm, an SNN based on the proposed algorithm for mobile robot obstacle avoidance is designed. The task of the SNN is to learn to avoid obstacles on the left or right side. To protect the robot from damage, a structure to control the braking is included in the network. The architecture of the whole SNN can be divided into two parts, the first part is related to obstacle avoidance and the second is related to brake. Next, two different parts of the SNN will be introduced separately and all the parameters that appeared in this section can be found in Table 2.

**Table 2:** The parameters used in the data transform.

**Parameter**	**Parameter description**	**Value**
*d*_*min*_	Minimum distance that can be measured	15 cm
*d*_*max*_	Maximum distance that can be measured	30 cm
*I*_0_	Init current of coding neuron	0.225 nA
*C*_*I*_	Conversion coefficient of distance	0.005 nA/cm

**Figure 4:**
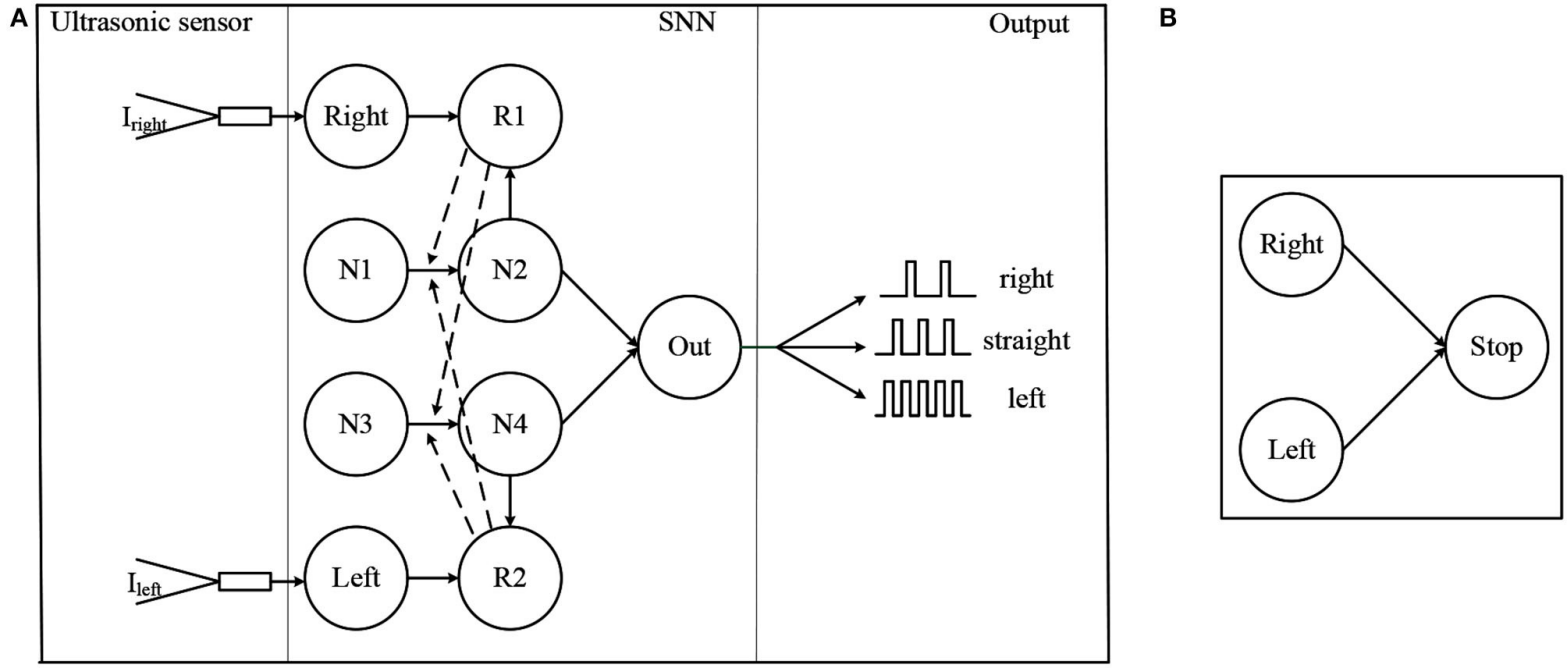
The proposed SNN, where **(A)** is the obstacle avoidance part and **(B)** is the brake part.

The first part is the obstacle avoidance architecture of SNN, as shown in Figure 4A. The solid arrow in the SNN module indicates the presence of a synapse, while the dashed arrow indicates that the reward signals are released into the synapse. The role of neurons Right and Left is to convert the distance data measured by ultrasonic sensors into spike trains. For convenience, these two neurons are the IF model, and the remaining neurons are the LIF model. *I*_*right*_ and *I*_*left*_ are the injected current and can be calculated by


(11) $$\begin{array}{l} \{\begin{array}{c} I_{left}=I_{0}-C_{i}d_{left}\\ I_{right}=I_{0}-C_{i}d_{right} \end{array}, \end{array}$$

where *I*_0_ and *C*_*i*_ are the constant, *d*_*left*_ and *d*_*right*_ are the distance information measured by the left and right ultrasonic sensors and are clipped in [*d*_*min*_, *d*_*max*_]. Neuron Right or Left fire spikes about 25 Hz when *d*_*left*_ or *d*_*right*_ is *d*_*max*_ and fire about 50 Hz when *d*_*left*_ or *d*_*right*_ is *d*_*min*_. Neurons R1 and R2 are dopaminergic neurons and are responsible for the release of the reward signals. Neurons N1, N2 and Neurons N3, N4 are two pairs of neurons implicated in information processing. These two pairs of neurons are used to simulate the cortical neurons in the brain (Lobov et al., 2020). Neurons N1 and N3 have no external input and they fire spikes at 25 Hz at all times. Neuron Out is responsible for decision making. The synapse between N2 and Out is inhibitory while excitatory between N4 and Out. The decision making of the SNN is determined by spike frequency of neuron Out. Robot keeping straight when neuron Out is firing from 8Hz to 18Hz. When the frequency is greater than 18Hz, the robot turns left. When the frequency is less than 8Hz, the robot turns right.

The following is a brief description of how the network works. When there is no obstacle, the negative current provided by N2 and the positive current provided by N4 remains the same, so the firing frequency of neuron Out is kept between 8 and 18 Hz. As the left side gradually approaches the obstacle, the frequency of spikes fired by the Left increases, leading to a higher level of the membrane potential of the dopamine neuron. In this case, R2 releases the reward signal when a spike is fired by N4. Since the spikes fired by N4 cause the release of the reward, the weight between N3 and N4 increases while the weight between N1 and N4 does not (The weight changes only when both the eligibility trace and the reward signal are present). As a result of increasing weights, the frequency of N4 and Out firing spike increases, which leads the network to make the decision to turn right. The increase in weight also leads to an increase in the γ in Equation 8. After avoiding the obstacles, the frequency of spike fired by Left decreases and R2 stops releasing the reward signal. The weight between N1 and N4 gradually decays back to its initial value and the network also starts to return to its original state.

From the above process, it can be seen that the function of the weight decay in Equation 8 is to restore the weight to its initial value, once the robot has completed obstacle avoidance. Without this, the network will keep the decision to turn right after the decision is made. This problem of not being able to turn straight ahead after turning right has appeared in the Lobov et al. (2020), and the weight decay can solve this problem. However, the decay of the weights over time means that the network cannot remember the experience and is not capable of learning. Therefore, in the improved algorithm, the learning rate γ is designed to be the same form as the weight in the original reward modulated STDP learning algorithm. This leads to a shift in the learning parameters of the network from the weights to the rate of change of the weights. Since the upper and lower parts of the network are symmetrical, the network works exactly the same as the left side when an obstacle appears on the right side.

The second part of the SNN architecture is shown in Figure 4B, it is a simple network composed of three neurons. All synapse between them is excitatory. Neurons Left and Right are mentioned above, and Stop is the neuron that controls the robot to move or stop. If neurons Left and Right both fire spikes near 50 Hz, in other words, obstacles appear on both sides of the robot, neuron Stop will fire spikes, then the robot stops moving. Otherwise, neuron Stop will not fire spikes, and the robot keeps straight.

### 2.7. Hardware Implementation

**Figure 5:**
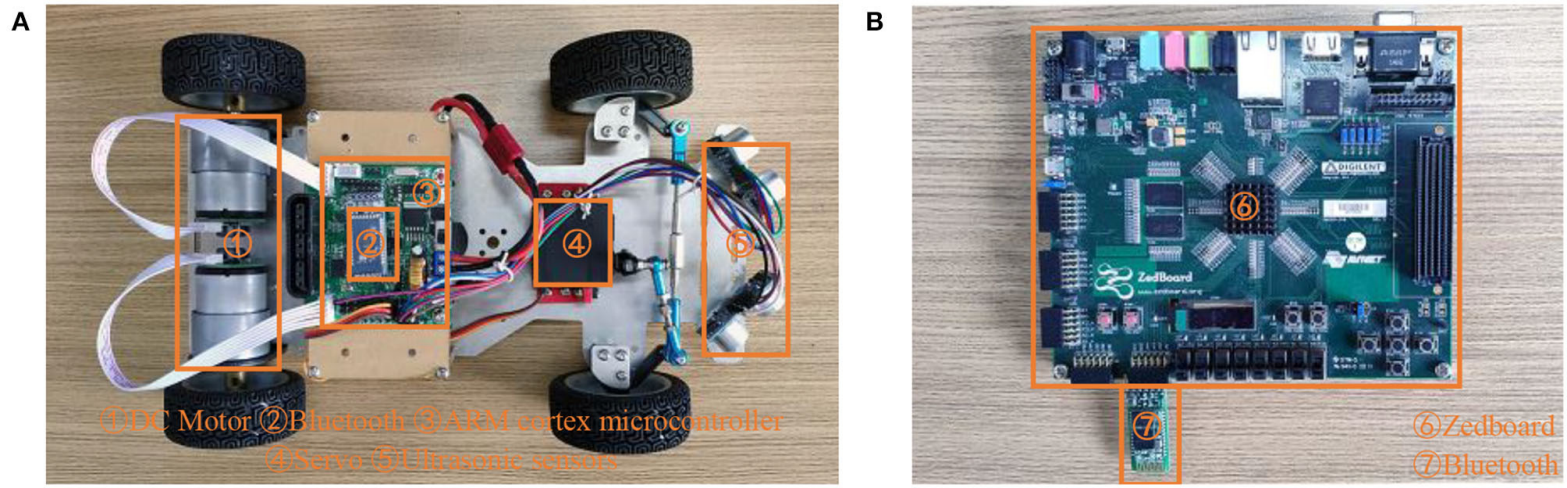
The hardware platform to implement the proposed SNN, where **(A)** is the mobile robot and **(B)** is the Zedboard development kit.

In this part, the hardware platform of the mobile robot is introduced. The platform can be divided into two parts. The first part is the torso of the robot, as shown in Figure 5A, an ARM Cortex microcontroller is used for controlling all the actions of the robot. The robot is equipped with two ultrasonic sensors with a 60° at the front, this makes the robot able to detect obstacles ahead. Moreover, the robot is also equipped with two motors and one servo, which are used to drive the robot and control the direction. The second part is a Zedboard development kit, which is responsible for decision making and can be considered the brain. All the SNN components proposed in this article are implemented in this part, as shown in Figure 5B. Two parts of the robot are communicated *via* Bluetooth interface and exchange data every 100 ms. The robot needs to send distance data measured by two ultrasonic sensors to the Zedboard, then the SNN in Zedboard analyses these data and sends the command to the robot. There are four kinds of commands, namely, keep straight, turn left, turn right, and stop.

**Figure 6:**
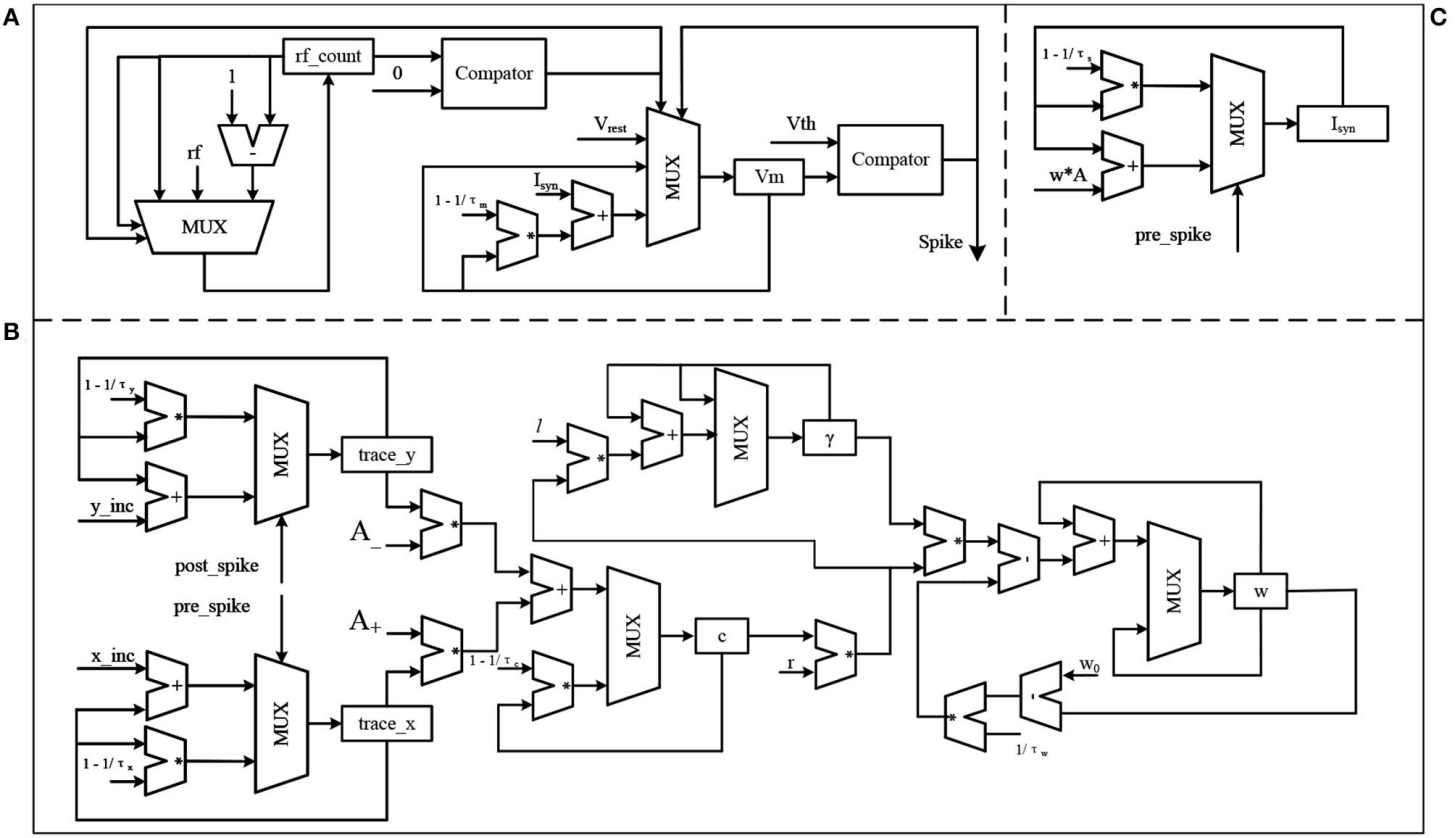
The hardware implementation of the main components in the proposed SNN, where **(A)** is the LIF neuron model, **(B)** is the α synapse, and **(C)** is the proposed algorithm.

Figure 6 shows the hardware implementation of the main components of the proposed SNN. The first is the LIF neuron, as shown in Figure 6A. This model can be divided into two parts, the refractory period counting part and the membrane potential update part. The register *rf*_*count* is used to represent the state of the neuron, *rf*_*count*>0 means that the neuron is in the refractory period and *rf*_*count* = 0 means the opposite. At each SNN time step, if a spike is fired by this neuron, the register *rf*_*count* is set to the *rf*, otherwise, the *rf*_*count* will be subtracted by 1 until it reaches 0. The register *V*_*m*_ stores the membrane potential of the neuron, the neuron will fire a spike when *V*_*m*_ > *V*_*th*_ and set the value of *V*_*m*_ to *V*_*rest*_. If *V*_*m*_ < = *V*_*th*_, *V*_*m*_ will be updated according to the injected current *I*_*syn*_ and its current value. The second part is the α synapse, as shown in Figure 6B. The function of this part is that at each SNN time step if a spike is fired by the pre-synaptic neuron, w*A is accumulated in the register *I*_*syn*_, otherwise, the value of the register *I*_*syn*_ decays. In this part, w is the synapse weight, A is a constant and its value is 0.5. The third part shows the proposed algorithm, as shown in Figure 6C. In this part, the hardware structure remains essentially the same as in the previous equations, *trace*_*y* and *trace*_*x* are the traces left by the post-synaptic and pre-synaptic spike, respectively, corresponding to *y*(*t*) and *x*(*t*) in Equation 5. Similarly, γ, *r*, *c*, and *w* are corresponding to γ(*t*), *r*(*t*), *c*_*ij*_(*t*), and *w*_*ij*_ in Equation 8. Based on the above models, this article uses a direct connection for the SNN implementation shown in Figure 4, which means that each unit in the SNN has a corresponding hardware circuit. The hardware resource cost of the SNN implemented based on the above model is shown in Table 3. The frequency of the system is 50 MHz, and the simulation data used in the proposed SNN is represented by 24-bit fixed-point numbers.

**Table 3:** The hardware resource cost of the proposed SNN.

**Module**	**LUT**	**FF**	**DSP**
LIF neuron model	240	38	0
α synapse	250	33	0
The proposed algorithm	1331	265	3
The proposed SNN	7792	1395	10
The whole system	7892	1524	10

## 3. Results

This section gives two experimental results of the mobile robot. The first experiment introduces how the robot learns to avoid obstacles by using the proposed algorithm, and the second is the obstacle avoidance performance test. In the second test, several obstacles are placed in the forward path of the robot to evaluate the avoidance performance of the robot.

### 3.1. The Demonstration of the Proposed Learning Algorithm

**Figure 7:**
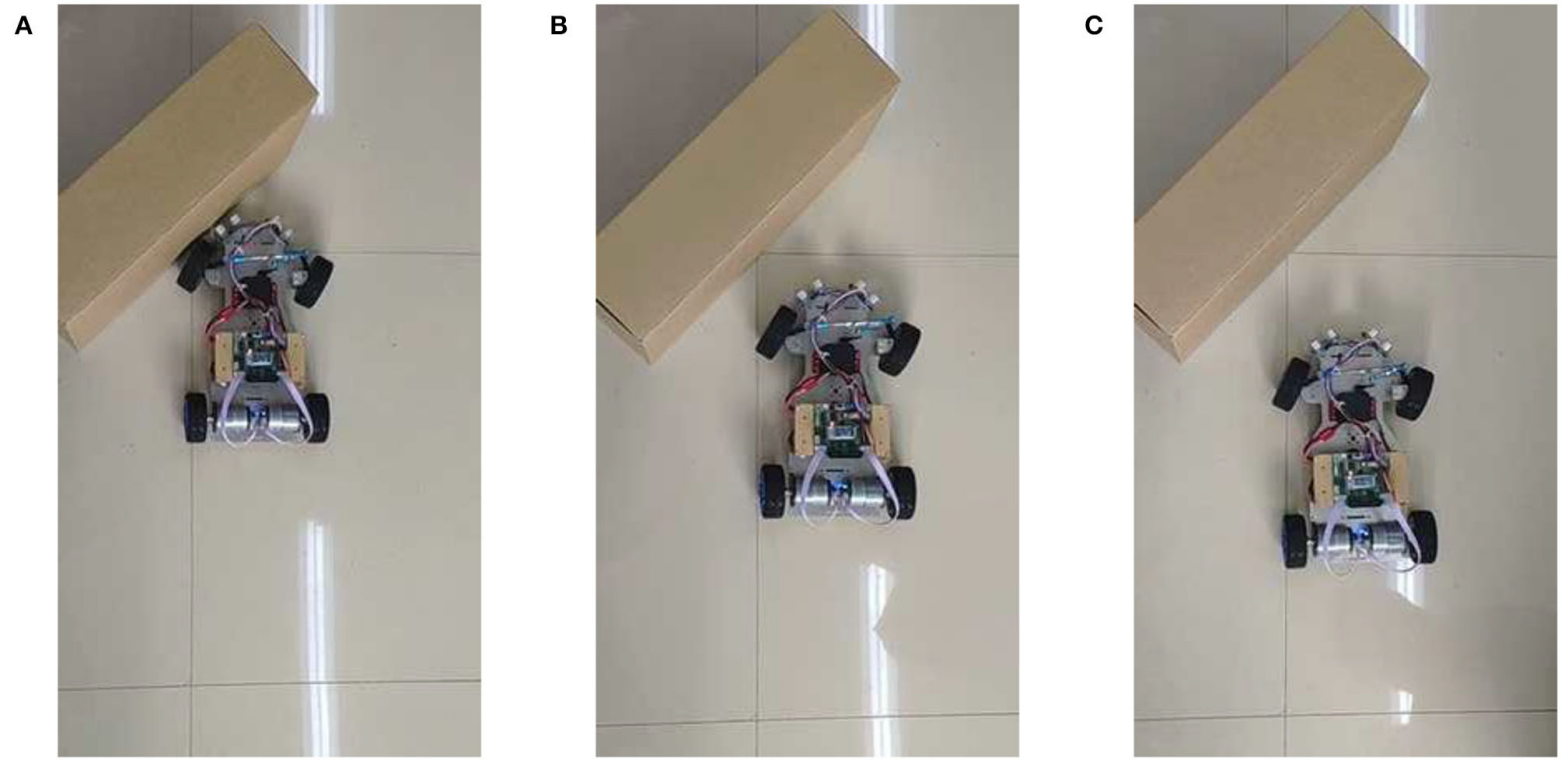
The moment when the mobile robot makes a steering decision in three learning, where **(A–C)** are the first learning, the second learning, and the third learning, respectively.

In this part, the mobile robot will be placed in an environment with only one obstacle and then learn to avoid obstacles. Since the learning process for obstacle avoidance on the left and right sides is similar, results for only the left side are provided here. The learning process on the left side is shown in Figure 7. These pictures capture the moment when the robot decides to turn right.

In the learning process, the robot is far from the obstacle at the beginning, so it keeps straight. As the robot gets close to the obstacle, the robot decides to turn right. However, in the first learning process, the robot is not skilled at avoiding an obstacle, so it cannot avoid the obstacle. As shown in Figure 7A, when the robot decides to turn right, it has already hit the obstacle, so it is a failed obstacle avoidance. In the second learning, its obstacle avoidance ability has been improved. As shown in Figure 7B, when the robot decides to turn right, there is still a certain distance between it and the obstacle. This is successful obstacle avoidance. In the third learning, the obstacle avoidance skill of the robot is further improved. As shown in Figure 7C, the robot decides to turn right even further away from the obstacle. This makes the robot more likely to avoid obstacles.

**Figure 8:**
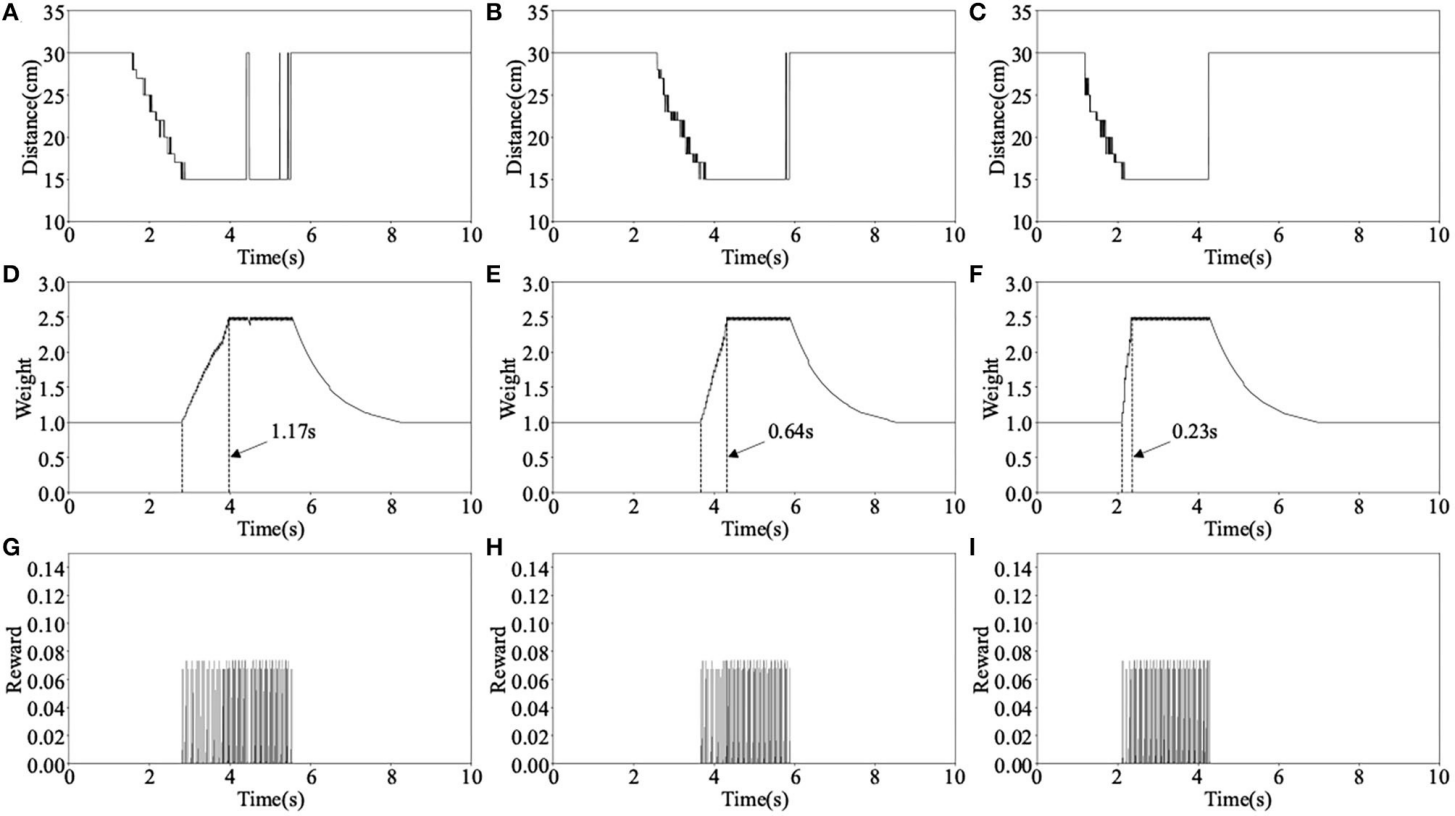
The main parameters change in the first experiment, where **(A–C)** are the distance data measured by the left ultrasonic sensor, **(D–F)** are the synaptic weight of neurons N3 and N4, and **(G–I)** are the reward signals released by neuron R2.

The main parameters change during the above learning process are shown in Figure 8, where Distance is the distance data measured by the left ultrasonic sensor, Weight is the weight between neuron N3 and N4, and Reward is the reward signals released by dopaminergic neuron R2. The three columns of data in Figure 8 correspond to the learning process in Figure 7. Take the first learning process as an example, when distance measured by ultrasonic sensor reaches the minimum value, the reward signals are released and weight begins to increase, as shown in Figures 8A,D,G. When the weight reaches near the maximum value, the robot decides to turn right, as shown in Figure 7A. It can be seen from Figure 8D that the time it takes for the weight to rise from the initial value to the maximum is 1.17 s, this is the reason why the obstacle cannot be avoided. However, during the second and third obstacle avoidance, the accumulated experience in obstacle avoidance makes it only need 0.64 and 0.23 s, as shown in Figures 8E,F. After each learning process, the learning rate of the corresponding synapse has increased. A higher learning rate means more proficiency in obstacle avoidance and less decision time.

### 3.2. Multiple Obstacle Avoidances

**Figure 9:**
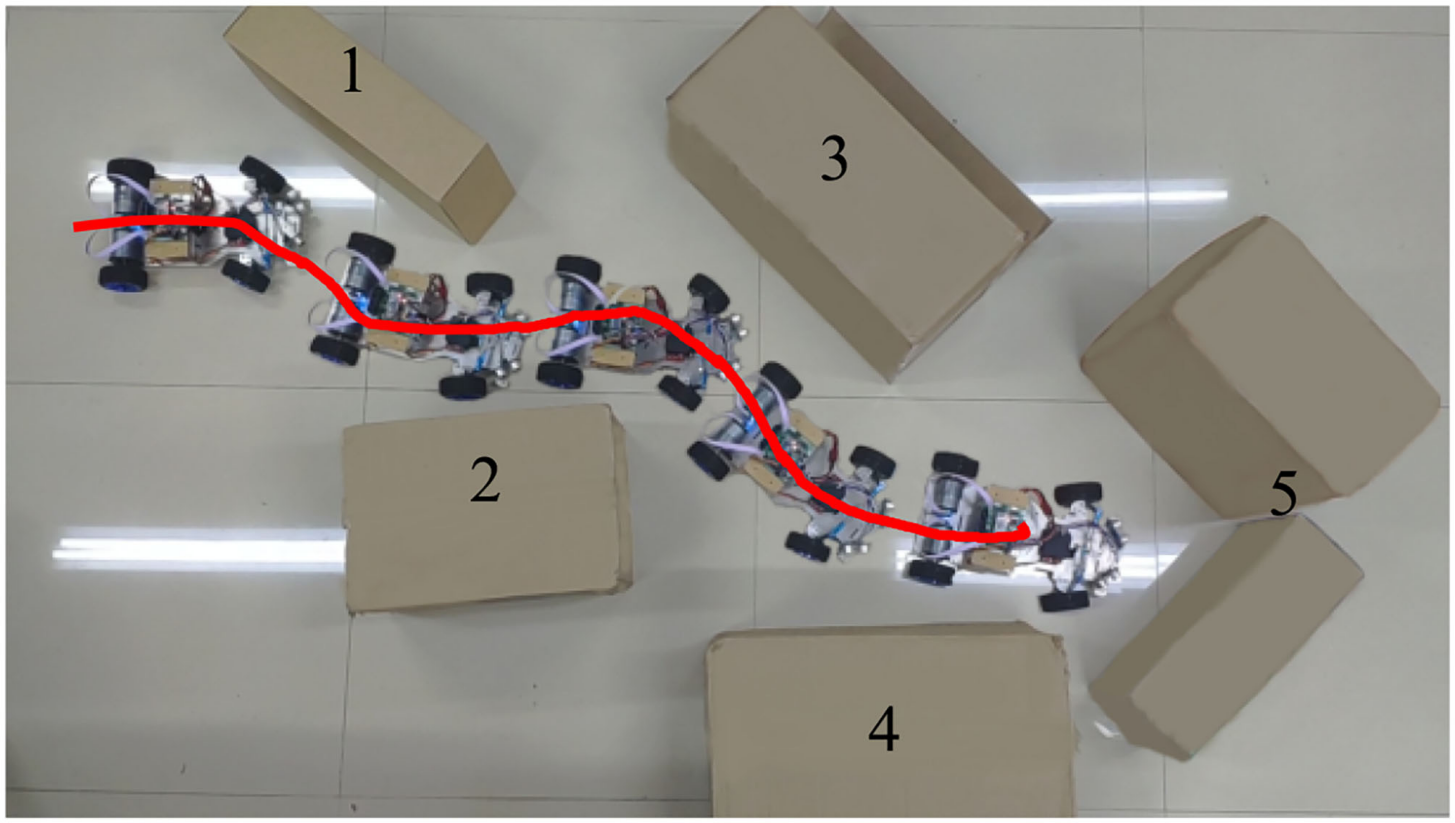
The travel route of the mobile robot when avoiding obstacles.

In this part, the robot has gone through sufficient learning and the obstacle avoidance performance of the mobile robot is tested in Section 4.1. The task of the mobile robot is to avoid several successively placed obstacles and then stop in front of the last obstacle. As shown in Figure 9, obstacles 1~4 all consist of a single carton, while obstacle 5 consists of two. This placement will let both left and right ultrasonic sensors detect the obstacle when the mobile robot reaches obstacle 5.

**Figure 10:**
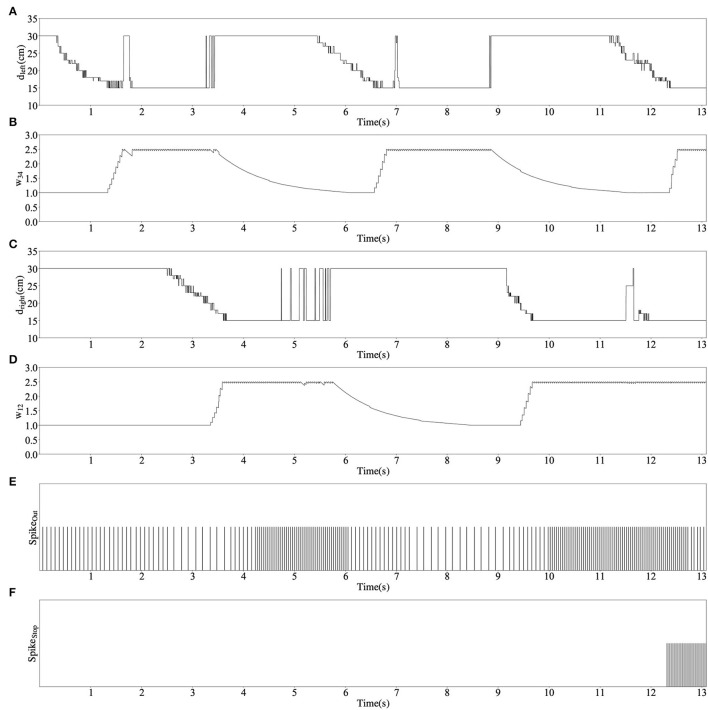
Parameters' changes in the obstacle avoidance test, where **(A)** is the distance data measured by left ultrasonic sensor, **(B)** is the synaptic weight *w*_34_, **(C)** is the distance data measured by the right ultrasonic sensor, **(D)** is the synaptic weight *w*_12_, **(E)** is the spike fired by neuron out, and **(F)** is the spike fired by neuron Stop.

The red solid line in Figure 9 shows the trajectory of the robot's movement. It can be seen from the trajectory that the robot always keeps a certain distance from obstacles during obstacle avoidance. Figure 9 also shows how the robot act when encountering each obstacle. This result shows that the robot has completed its intended task. The changes in the main parameters in this obstacle avoidance process are given in Figure 10. All these data are collected in real-time from the Zedboard.

In the beginning, no obstacles are detected, the spike frequency of neuron Out is about 12 Hz and the robot keeps straight. At approximately 0.3 s, the robot encounters obstacle 1 and weight *w*_34_ begins to rise under the action of the reward signals. As a result, the spike frequency of neuron Out gradually decreases, and the robot turns right. Afterward, at approximately 2.5 s, the robot immediately encounters obstacle 2, weight *w*_12_ begins to rise at about 3.3 s while *w*_34_ decays to its initial value at about 6 s. This causes an increase in spike frequency of neuron Out and the robot turn left. The data's changes in Obstacles 3 and 4 are the same as 1 and 2's. Note that in the processes of avoiding obstacles 1~4, neuron Stop does not fire spikes, so the robot always keeps moving. However, when the robot encounters obstacle 5 at 11 s, both left and right ultrasonic sensors detect the obstacle, hence neuron Stop starts firing spikes and the robot stops moving.

## 4. Discussion

For the problem of obstacle avoidance, a rule-based design may be simpler. However, to explore how to build more intelligent, biologically inspired robots based on SNN models, the evaluation of the system performance should be based on biological behaviors. Therefore, we use a qualitative analysis (Mayer, 2002) to evaluate the system by classifying its learning types. According to Mayer (2002), there are two key factors of learning, namely retention and transfer. Retention is the ability to remember material and transfer is the ability to use what was learned to solve new problems. Depending on the presence or absence of retention and transfer, learning can be classified as no learning, rote learning, and meaningful learning. Meaningful learning has both retention and transfer while rote learning only has retention, and no learning has neither retention nor transfer. Therefore, within this scope of definitions, the experiment in Section 3.1 shows that the robot can successfully avoid the obstacle with a fixed position on the left side, i.e., the robot has the ability of retention. The experiment in Section 3.2 shows that after learning, the robot can successfully avoid multiple obstacles placed on both sides that it has never seen before, i.e., the robot has the ability of transfer. Therefore, the robot designed in this study successfully implements meaningful learning. All these behaviors only require a network with ten neurons and a learning rule, which demonstrates that even a simple neural network can show behaviors that look remarkably sophisticated to outside observers (Reynolds, 1987).

There have also been previous articles exploring the use of SNNs to model biological behavior which is listed in Table 4. In Lobov et al. (2020), an SNN model implements associative learning through an STDP algorithm and is used for controlling the mobile robot. The mobile robot exhibits successful learning at the behavioral level in the form of classical and operant conditioning. However, that mobile robot is only able to avoid one side of the obstacles after learning and if the environment changes, the robot needs to relearn. This suggests that although the robot learns the behavior of obstacle avoidance, it cannot extend it to a more complex environment. Therefore, the robot in Lobov et al. (2020) could be considered only performed rote learning. In Blum et al. (2017), a robotic vehicle equipped with a dynamic vision sensor is designed to solve obstacle avoidance and target acquisition problem. The experiment results show that the robot can achieve the expected results in a variety of test environments, demonstrating the potential of SNN for solving complex problems. However, the ability of the robot to avoid obstacles and acquire targets is innate, i.e., it has these abilities without a learning process. The robot does not have the ability to learn, so there is no retention and transfer process (no learning). In Shim and Li (2017), a multiplicative reward modulated STDP is proposed and applied to the mobile robot collision avoidance. The mobile robot can reach the target location without collision, and it needs only 20 training trails. The mobile robot designed by Tang and Michmizos (2018) uses an SNN to learn how to explore an unknown environment. However, these two robots are also limited by the environment and need to relearn when the environment changes, so they can only perform rote learning. In addition, all those reported performances are based on software simulation.

**Table 4:** Comparisons with previous studies.

**Approach**	**Function**	**Learning algorithm**	**Learning type**	**Hardware platform**
Lobov et al. (2020)	Mobile robot which can learn to avoid obstacles	STDP	Rote learning	PC and ARM Cortex microcontroller
Blum et al. (2017)	Mobile robot which can avoid obstacles and acquire target	NA	No learning	Neuromorphic chip and ARM Cortex microcontroller
Shim and Li (2017)	Mobile robot which can learn to avoid obstacles and reach a specific location	Reward modulated STDP	Rote learning	Software simulation
Tang and Michmizos (2018)	Mobile robot which can learn to explore in real word	STDP	Rote learning	Software simulation
This work	Mobile robot which can avoid obstacles	Reward modulated STDP	Meaningful learning	FPGA and ARM Cortex microcontroller

In addition to the ability to learn, the hardware platform is also an important part of the implementation of neuroanimats. Due to the large number of computational resources needed to be spent on simulating the behavior of biological neurons, Tang and Michmizos (2018) and Lobov et al. (2020) both use PC for the implementation of the proposed SNN. However, the large size and power consumption of PC are not suitable for neuroanimats, especially when neuroanimats need to explore the environment. A neuromorphic processor like Blum et al. (2017) used is another option. But as mentioned in the article, all neurons are used and it is impossible to extend their work with additional behaviors. This shows that the fixed architecture neuromorphic processors are not flexible enough in the research phase. Therefore, the combination of microcontroller and FPGA may be more suitable for neuroanimat researchers.

## 5. Conclusion

A biologically inspired autonomous learning algorithm is proposed in this paper to explore the learning mechanism in the brain. Based on this algorithm, an SNN with special dopaminergic neurons is designed. The stimulation of dopaminergic neurons is related to environmental changes, and the reward signals released by dopaminergic neurons determine which actions the robot takes. Experimental results show that the mobile robot can successfully solve the obstacle avoidance problem and simulates the learning process of animals well. With only a few learning sessions, the mobile robot can avoid obstacles on one side and apply its obstacle avoidance ability to more complex environments. Even if the robot does not have any prior knowledge about the test environment, it can avoid all obstacles without collision. Besides, the FPGA hardware system is designed to accelerate the proposed SNN, which not only improves the performance of real-time data processing of the robot but also provides a hardware platform for further expansion. Future study is planned to apply the proposed algorithm to solve more complex problems of robot control, such as object tracking and route planning.

## Data Availability Statement

The original contributions presented in the study are included in the article/supplementary material, further inquiries can be directed to the corresponding author/s.

## Author Contributions

JL, YH, RY, and YL developed, implemented, and evaluated the neural network algorithm. JL and YH wrote and revised the manuscript. HL, YW, SY, and XD analyzed the performance of the proposed network and reviewed the manuscript. All the authors contributed to the article and approved the submitted version.

## Funding

This research was partially supported by the National Natural Science Foundation of China under Grant No. 61976063 and the Guangxi Natural Science Foundation under Grant No. 2022GXNSFFA035028.

## Conflict of Interest

The authors declare that the research was conducted in the absence of any commercial or financial relationships that could be construed as a potential conflict of interest.

## Publisher's Note

All claims expressed in this article are solely those of the authors and do not necessarily represent those of their affiliated organizations, or those of the publisher, the editors and the reviewers. Any product that may be evaluated in this article, or claim that may be made by its manufacturer, is not guaranteed or endorsed by the publisher.
